# The Chile BiLS case–control study of gallbladder cancer

**DOI:** 10.1186/s13690-026-01977-1

**Published:** 2026-06-06

**Authors:** Jill Koshiol, Vanessa Van De Wyngard, Ilona Argirion, Claudia Marco, Matías A. Pozo Diaz, Ian N. Reyes Romero, Paz Cook, Ruth M. Pfeiffer, Noldy Mardones, Karie Medina, Vanessa Olivo, Karen Pettit, Raúl Sanchez, Miguel Villaseca, Enrique Bellolio, Hector Losada, Juan Carlos Roa, Allan Hildesheim, Marco Aresse, Catterina Ferreccio, Juan Carlos Araya

**Affiliations:** 1 Division of Cancer Epidemiology and Genetics, National Cancer Institute, Rockville, MD USA; 2 Facultad de Medicina, Pontificia Universidad Católica de Chile, Santiago, Chile; 3 Department of Human Science, School of Health, Georgetown University Medical Center, Washington, DC USA; 4 Department of Epidemiology, Gillings School of Global Public Health, University of North Carolina at Chapel Hill, Chapel Hill, USA; 5 Hospital Dr. Hernan Henríquez Aravena, Temuco, Chile; 6 Westat, Inc., Rockville, MD USA; 7 Facultad de Medicina, Universidad de La Frontera, Temuco, Chile; 8 Center for Cancer Prevention and Control (CECAN), Santiago, Chile; 9 Agencia Costarricense de Investigaciones Biomedicas, San Jose, Costa Rica; 10 Instituto de Salud Pública de Chile, Santiago, Chile

**Keywords:** Gallbladder cancer, High-grade dysplasia, Etiology, Molecular epidemiology, Early detection

## Abstract

**Background:**

Gallbladder cancer (GBC) is highly fatal and, unlike most cancers, is more common in women than in men. Most GBC cases have gallstones, but most people with gallstones do not develop GBC. Thus, a critical question is what drives the risk of GBC in the presence of gallstones.

**Methods:**

We designed a case–control study to complement the Chile Biliary Longitudinal Study (Chile BiLS) cohort, enriching the number of GBC cases and enhancing our ability to evaluate risk factors for GBC. Starting in July 2022, we began to recruit non-cohort prevalent (diagnosed between January 1, 2016, and July 18, 2022) and incident (diagnosed on or after July 19, 2022) GBC cases, as well as patients with high-grade dysplasia (HGD), in a high-risk area of Chile. Individuals with GBC or HGD are considered cases (HGD +). We are matching gallstone cholecystectomy patients to cases at an approximate 1:1 ratio. We also include unmatched controls (adjudicated) who were initially suspected of having cancer but had benign findings on gallbladder pathology. If a case or control is deceased, we conduct proxy interviews to maximize the potential for detailed epidemiological data collection. Through August 31, 2025, we recruited 196 prevalent and 117 incident HGD + cases. Of these cases, 189 (96%) prevalent and 108 (92%) incident cases were diagnosed with GBC. All prevalent and 98.3% of incident cases have matched controls. Participation rates are 70.8% for HGD + cases and 67.8% for matched controls.

**Discussion:**

The Chile BiLS case–control study is conducted in an area with high risk of GBC and a high proportion of people with Mapuche Amerindian ancestry. This study will provide important insights into the factors associated with GBC among people with gallstones. Here, we provide a thorough description of the design of the study, field procedures, and biological resources, as well as research opportunities, which will enable a more complete picture of the etiology of GBC by combining epidemiological, molecular, digital imaging, and clinical data. This study represents a powerful resource for the identification of new targets for cancer prevention and treatment, which are particularly needed in populations at high risk of GBC.

**Supplementary Information:**

The online version contains supplementary material available at 10.1186/s13690-026-01977-1.

**Table:** 

Text box 1. Contributions to the literature
• Gallbladder cancer is understudied and constitutes a major public health burden in some regions and subpopulations.
• Well-characterized epidemiologic studies in high-risk regions are needed to improve our understanding of the causes of this disease and to identify targets for prevention, early detection, and treatment.
• The Chile BiLS case–control study provides a unique opportunity to explore risk factors for gallbladder cancer and high-grade dysplasia in individuals with gallstones.

## Background

Gallbladder cancer (GBC) is highly fatal, with an overall 5-year survival of less than 20% [1, 2]. Although rare in much of the world, GBC is a leading cause of cancer death in women in some parts of the world, such as southern-central Chile and northeastern India [3–5]. GBC risk is particularly high in certain subpopulations, such as indigenous populations and Hispanic individuals [5]. Even in low-risk countries, such as the United States (US), incidence rates are increasing in Black individuals and are projected to continue to increase in Black men and women [6]. Current treatments are insufficient; newly approved drugs mainly improve intrahepatic bile duct cancer survival, with no impact on GBC [7, 8]. A concerted effort is needed to improve the understanding of GBC epidemiology and its molecular underpinnings, which could help identify targets for prevention, early detection, and improved therapeutics.

The majority of GBC cases are associated with gallstones, approaching 90% of GBC cases in some high-risk areas [9]. Epidemiological studies have found that gallstones are associated with an increased risk of developing GBC [10, 11] and that the risk increases with longer duration [12–14] and larger stone size or volume [10, 15–18]. However, only about 1% of people with gallstones develop GBC [19], suggesting that additional co-factors are needed to drive progression to GBC after gallstones develop. Other than gallstones, the etiology remains largely unclear, and molecular analyses lag behind.

We previously described a cohort study of women with gallstones designed to address this question in a high-risk area of Chile: the Chile Biliary Longitudinal Study (Chile BiLS) [20]. To enrich the cohort, we developed an accompanying case–control study. This study provides additional GBC cases with epidemiological and clinical data, blood collection, and complete sampling of the gallbladder, with digital imaging of the resulting hematoxylin and eosin (H&E) sections.

Here, we describe the Chile BiLS case–control study and its integration with the cohort component, which together provide a unique opportunity to explore risk factors for gallbladder cancer and its precursors in individuals with gallstones. This approach also aims to identify new targets for prevention and treatment of this highly fatal and understudied cancer. By increasing the number of cases, the study will offer valuable insights into the etiology of gallbladder cancer.

## Methods/design

### Study design

We are conducting the Chile BiLS case–control study in the southern part of the Araucanía region (Cautín Province), the same region as the Chile BiLS cohort [20]. This region is in the southern-central part of Chile and has high rates of GBC and a large proportion of individuals with Mapuche Amerindian ancestry [21]. Greater than 90% of the Chilean population in this region is covered by the public health system [22]. Recruitment began in July 2022, with 1) retrospective identification of cases and controls who were diagnosed with cancer or had cholecystectomy between January 1, 2016, and July 18, 2022 (prevalent cases and controls) and 2) prospective identification of cases and controls who were diagnosed with cancer or had cholecystectomy on or after July 19, 2022 (incident cases and controls).

The study was approved by the Pontificia Universidad Católica de Chile ethical review board (Santiago, Chile) and the Scientific Ethics Committee, Araucanía Sur Health Service (Araucanía, Chile). All study participants or, if the participant was deceased, their proxies provide written informed consent.

Men and women aged 18 years or older who are residents of Cautín Province, La Araucanía Region, at the time of diagnosis or selection as a control are eligible for recruitment. Participants who are alive at the time of recruitment must have the physical and mental capacity to consent. Participants who are dead must have a proxy with the physical and mental capacity to consent. Dead participants also have to have gallbladder tissue available (at least one tissue block stored in the Hospital Dr. Hernán Henríquez Aravena (HHHA) Department of Pathology).

Participant identification and notification are based on an integrated system linking routine clinical practice with the research team, under appropriate ethical approvals allowing access to clinical records in participating hospitals. Through this system, potential cases and controls are identified from multiple clinical sources, including pathology records, oncology committees, and surgical schedules. Once a potential participant is identified, the local research team reviews clinical records to confirm eligibility criteria, vital status, and feasibility of contact. Subsequently, contact is initiated, typically by telephone followed by at home visits when necessary, to invite participation in the study. This approach enables timely identification, notification, and enrollment of both incident and prevalent participants.

**Fig. 1:**
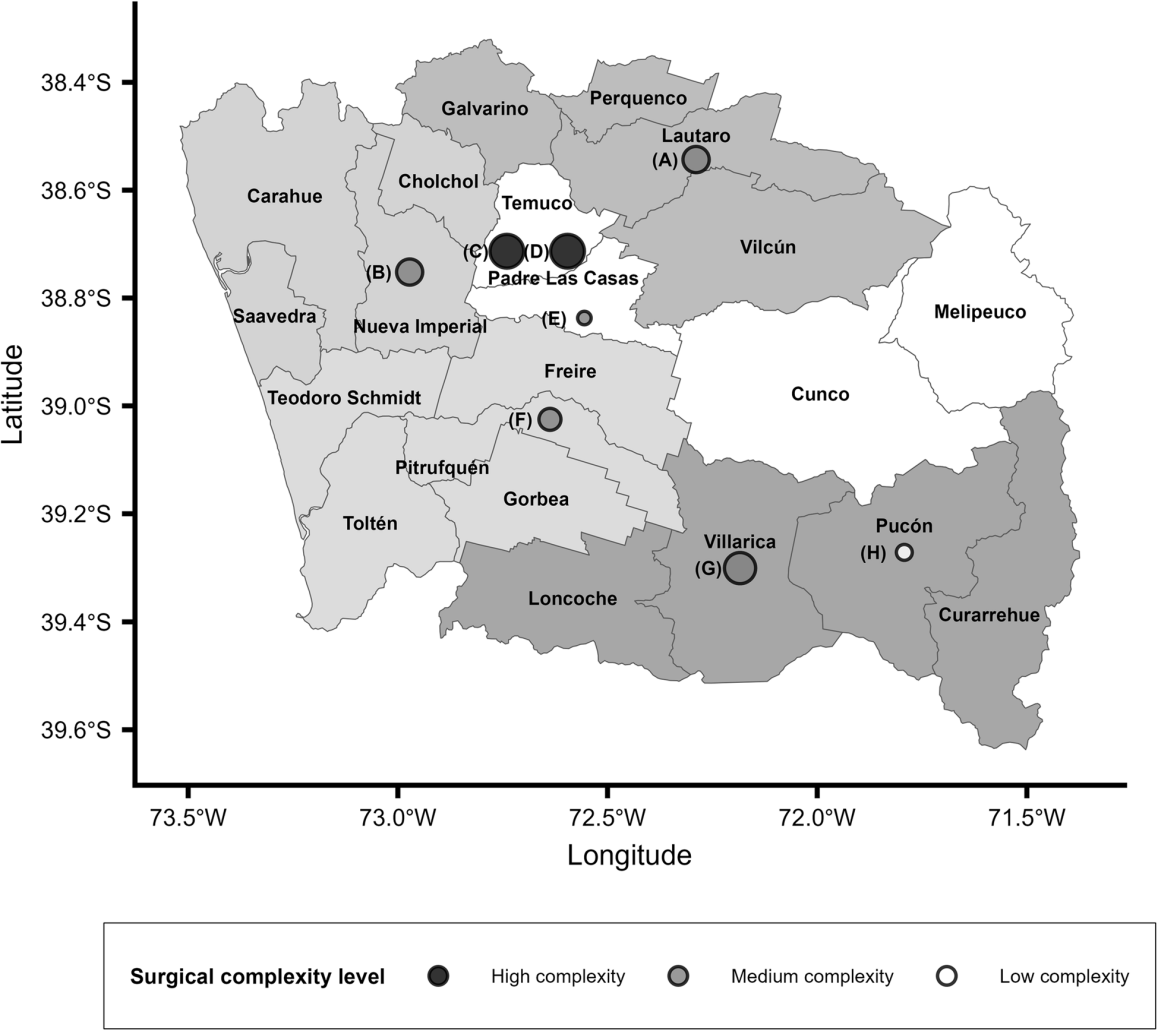
Geographical location of the health centers in the province of Cautín included in the case–control study. The grayscale shading indicates the counties that comprise the health coverage assigned to each health center. The size of the dots is proportional to the number of cholecystectomies performed in 2018 in the county where the health center is located, relative to the total number of cholecystectomies performed in the health coverage of that health center in that same year. **A** Dr. Abraham Godoy Peña Hospital. **B** Nueva Imperial Intercultural Hospital. **C** Hernán Henríquez Aravena Hospital (Regional Hospital of Temuco). **D** RedSalud Mayor Clinic of Temuco. **E** Complejo Asistencial Padre Las Casas. **F** Pitrufquén Hospital. **G** Villarrica Hospital. **H** San Francisco Hospital of Pucón

After confirming eligibility, the enrollment visit is typically conducted in a mobile clinic outside of the participant’s home or workplace; occasionally it takes place at the study clinic or a hospital. The hospitals participating in the case–control study include Complejo Asistencial Padre Las Casas, Clínica RedSalud Mayor Temuco (a private hospital), Hospital Hernán Henríquez Aravena de Temuco, Hospital de Lautaro, Hospital Nueva Imperial, Hospital de Pitrufquén, Hospital de Pucón, and Hospital de Villarrica; in total, these represent 8 out of 10 hospitals that conduct cholecystectomies in Cautín Province (Fig. 1).

The HHHA Pathology Department reviewed about 40 non-Chile BiLS cohort GBC cases per year between January 2016 and June 2021. We estimated that we could contact 75% of cases, i.e., 30 cases a year. Therefore, over 3.5 years of recruitment (from July 19, 2022, through March 3, 2026), we estimated we would enroll approximately 105 incident GBC cases outside the Chile BiLS cohort. In addition, we anticipated recruiting approximately 200 prevalent cases.

### Cases

Cases include individuals diagnosed with GBC or high-grade dysplasia (HGD +) between January 1, 2016, and July 18, 2022 (prevalent), or on or after July 19, 2022 (incident). Incident cases are identified through multiple sources (e.g., electronic medical records (EMRs), discharge registries, and specialist referral records). Once a participant is enrolled, the study coordinator reviews the electronic clinical record to determine the diagnosis of the participant. When the diagnosis is not clear, but GBC is suspected, the participant is recruited as a case, and the final diagnosis is confirmed through expert review. The expert review involves analyzing the available clinical records from the time of cancer suspicion, including imaging exams, histopathology, tumor markers, specialist consultations, and cause of death (if applicable). In addition, the review generates an assessment of whether confidence in the participant's cancer diagnosis is high, medium, or low based on the available evidence.

Incident cases are identified through multiple sources. The HHHA pathology team alerts the field team when they issue an HGD + compatible histological diagnosis (i.e., a histological diagnosis “compatible”, “suggestive”, “probable” or “that favors” pancreatobiliary cancer or HGD). At HHHA, routine pathologic examination of cholecystectomy specimens begins with review of one 3 mm wide section of the gallbladder cut from the edge of the cystic duct to the fundus. If low-grade dysplasia or worse pathology is identified in the diagnostic section, the entire gallbladder is processed and reviewed, a process called full mapping [23]. When a pathologist requests full mapping, the pathology team notifies the field team of a suspected HGD + case through established study procedures as part of routine communication within the study workflow. The research team, in coordination with the study pathologist, reviews clinical and pathology information to confirm eligibility before obtaining contact details through authorized clinical records and inviting the patient to the study. Participants identified through full mapping request are considered cases pending the final histological diagnosis. In addition, the HHHA Oncology Committee sends a weekly list of individuals they reviewed with a GBC or compatible clinical diagnosis. The field team also reviews the daily surgical schedules for all surgical hospitals in the study area to identify individuals scheduled for a procedure due to an indication of GBC. Furthermore, the field team conducts weekly visits to the surgical service of the regional hospital. Finally, the field team offers hepatobiliary ultrasound exams to family members of participants as a benefit for their participation. Abnormal images are reviewed by the study radiologist, who refers the individual to the hospital for further evaluation if necessary. Prevalent cases were identified similarly through the HHHA Pathology Department and Oncology Committee.

All potential cases (incident and prevalent) are enrolled when the suspicion of HGD + is first identified, and diagnosis is confirmed through review of electronic health records. While cases can be identified through multiple sources, the HHHA Pathology Department has been the primary source for 74.8% of cases (N = 234) to date. Cases identified through HHHA Oncology Committee account for 16.6% of cases (N = 52). Twenty-six cases (8.3%) have been identified through review of the daily surgical schedules or visits to the surgical service of the regional hospital. One case (0.3%) has been identified through study ultrasound.

The date of diagnosis is defined as the date of cholecystectomy for incidental cases (i.e., those detected through cholecystectomy) and as the date of the first clinical study (e.g., ultrasound) that led to diagnosis through the report of a suspicious lesion for clinical cases. Pathology diagnoses are determined through review by the HHHA pathology team, which always includes the pathology department head or deputy department head. Tumor histology is determined through pathological review. Staging follows the American Joint Committee on Cancer (AJCC) TNM system, which determines the extent, or size, of the tumor (T), involvement of the lymph nodes (N), and metastasis to distant sites (M) [24].

### Gallstone controls

Cautín Province residents with a cholecystectomy between January 1, 2016, and July 18, 2022 (prevalent), or on or after July 19, 2022 (incident), and whose histological diagnosis is not HGD + are eligible as controls. Matched controls are selected for all participants with a suspected or confirmed HGD + diagnosis.

#### Matched controls

To identify incident matched controls, the field team reviews the surgical schedules we receive from seven public surgical hospitals and one private hospital, excluding individuals already enrolled in the Chile BiLS case–control or cohort. When an incident case is enrolled, the field team selects potential incident controls who match the date of birth (± 5 years) and sex (inferred by name) of the case and who had surgery on the day closest to the case’s diagnosis (i.e., the cholecystectomy date for cases identified surgery or the date of first suspicion of cancer for clinical cases). If more than one potential participant is available, one is randomly selected. We did not match on hospital to avoid over matching (e.g., on environmental factors or other characteristics that might vary by the location of the hospital).

People whose gallbladders were received at the HHHA pathology laboratory between January 1, 2016, and July 18, 2022, excluding Chile BiLS participants, were eligible to be prevalent matched controls. When a prevalent case is enrolled, the field team selects potential prevalent controls by starting with the month in which the corresponding case was diagnosed, identifying the first person who had cholecystectomy after the case and matches the date of birth (± 5 years) and sex. Upon finding a match, the team repeats the process to identify a second potential control in case the first cannot be enrolled.

After confirming sex, date of birth, and residential status through review of official records, the potential control is invited to the study. If the potential control refuses, the field team repeats the processes described above to identify a new potential control. If an enrolled control does not have blood collected due to refusal or failed blood draw attempt, the team collects saliva as a source of germline DNA and then selects an additional potential control by repeating the processes described above and continuing until a control with collected blood is enrolled.

#### Adjudicated controls (i.e., case-eligible at recruitment, non-case after diagnostic confirmation)

In addition to matched controls, we have controls of clinical interest. These include people who were recruited because they had an HGD + compatible histological diagnosis but that diagnosis was later ruled out. Since we automatically enroll all individuals with such diagnoses, these individuals were enrolled but not matched to a case.

### Strategies to improve participation

In order to maximize study participation, we offered to perform hepatobiliary ultrasound exams on both the participant and their immediate family members (i.e., parents, siblings, children) and secondary family members (e.g., aunts and uncles) if not cholecystectomized as a benefit for their participation. Ultrasound for family members began in January 2023. From January 2023 through August 2025, 1,705 abdominal ultrasounds were conducted on family members of study participants. Ultrasounds for family members are ongoing. The study team counseled family members with abnormal ultrasound findings and referred them to their original healthcare facility for follow-up to ensure prompt treatment, if necessary. The most common reasons for referral included liver disease, suspicion of cancer, liver or renal cysts, or abnormal ultrasound findings (Supplementary Table 1). In addition to the ultrasound exam, participants are given monetary compensation of CH$5,000 (approximately US$6) for the time invested.

Offering ultrasound to family members seems to be valuable for improving participation. Enrollments increased shortly after initiating ultrasounds for family members. The field team noted that family ultrasounds have become a key incentive to enroll case–control participants. Participants value this free preventive exam and accompanying report that they can take to their treating physician.

### Epidemiological and clinical data collection

All data are collected using REDCap electronic data capture tools hosted at Yale University [25, 26]. Forms used for macroscopic review, microscopic review, and description of cancer are initially collected on paper by the pathologists during their review. These forms are then entered into REDCap and double-checked for accuracy. All other forms (questionnaire, physical exam) are directly entered into REDCap.

The questionnaire includes questions on socio-demographics, medical history, gastrointestinal symptoms, weight history, reproductive factors, chronic medication use, smoking and alcohol consumption, diet, work history, pesticide exposure, physical activity, sedentary behavior, and family medical history. The baseline questionnaires for the cohort and case–control components of Chile BiLS are highly similar in structure and content. Most core variables are identical or closely aligned, which will facilitate harmonization. Proxy interviewees complete an abbreviated version of the subject questionnaire (Supplementary Table 2).

The physical exam includes height, weight, waist circumference, and hip circumference. If we cannot make these measurements (e.g., if the participant is bed-bound), we ask the participant for weight and height information or try to obtain them from clinical records. We measure blood pressure once during administration of the questionnaire.

For all GBC cases, we conduct clinical record reviews, including electronic and paper clinical records from one or more hospitals as determined necessary to obtain all relevant clinical data (e.g., diagnosis of gallstones and GBC, gallbladder disease symptoms and other medical conditions, pathology and imaging results, surgery, chemotherapy, and radiation). For GBC cases, information regarding treatment (radiotherapy and chemotherapy) and tests, imaging, or clinical indications leading to diagnosis are also collected. Death date and cause of death are obtained through linkage with the national death registry for all subjects who were deceased at detection and proxy consent was given. Death certificates are obtained from the Civil Registry and Identification Service of Chile. The national death registry is searched every six months to identify participants who have died since their enrollment (most recent linkage March 31, 2025), and death certificate information is obtained for them.

### Biospecimen collection

Blood collection includes one 10 ml red top tube for the collection of serum, one 10 ml lavender top EDTA whole blood tube for plasma and buffy coat, and beginning in March 2023, one 6 ml lavender top tube to provide more plasma to allow for assays requiring somewhat higher plasma volume. If blood cannot be collected, we collect 2 ml of saliva using an Oragene kit (DNA Genotek, Canada) to provide a source of germline DNA. Information regarding fasting time, medication taken one week prior to collection, date of last menstruation, and symptoms the day of collection is registered. Blood samples are processed at the study laboratory within 30 min of collection if possible and no more than four hours after collection. During this time, samples are kept refrigerated at 4º C. Plasma and serum samples are aliquoted into FluidX cryovials in 0.5 ml aliquots. Blood-product aliquots are stored in −80 °C freezers in Temuco, Chile, and aliquots assigned to the US are later transferred to the Pontificia Universidad Católica in Santiago, Chile, for storage until they are shipped to the US National Cancer Institute. Saliva samples are stored at ambient temperature until transferred to the National Cancer Institute.

Tissue specimen availability varies according to the type of participant and how the diagnosis was made. For incident cases and controls, the field team notifies the pathology team immediately after enrolling a participant to ensure that the pathology team conducts full mapping of the gallbladder for that individual if they went to surgery. The practice of studying the entire gallbladder of GBC cases predates the initiation of the case–control study. Thus, incident and prevalent cases who went to cholecystectomy should have full gallbladder mapping. Available tissue blocks are split evenly between Chile and the US. Cases without cholecystectomy but with biopsy should have the tissue block(s) corresponding to the fragments obtained from a biopsy (percutaneous, endoscopic, or surgical). Such biopsies are generally taken from the liver but can also include “unspecified biliary tract mass/lesion”. Cases without cholecystectomy or biopsy (i.e., identified through imaging) will not have tissue available. Prevalent controls should have the diagnostic block(s) available since they are selected from people who have had cholecystectomy, and the Pathology Department protocol has been to save the diagnostic blocks. All available tissue is processed using the data collection form that was employed for the Chile BiLS cohort.

### Digital H&E slide images

H&E slides are scanned by the Santiago team using an Aperio AT2 DX by Leica Biosystems (Nussloch, Germany). The scanned images are uploaded to Box Enterprise Plus and then imported into the HALO digital pathology platform (Indica Labs, RRID:SCR_018350). We plan to make digital H&E slide images from all available H&E slides.

### Analytic approaches for future analyses

We will use conditional logistic regression models to estimate odds ratios and 95% confidence intervals for associations with HGD + versus matched controls, possibly with further adjustments. We will compare results from conditional logistic models with results from unconditional logistic models adjusted for all matching factors, and if results are similar, we will present results from the unconditional models as the main analysis as they often provide smaller variance estimates. For comparisons of cases with adjudicated controls, we will use unconditional logistic regression. We will assess potential differences across case group by conducting sensitivity analyses 1) excluding HGD cases and 2) evaluating cholecystectomy detected and clinically diagnosed cases separately. Similarly, we will conduct sensitivity analyses excluding proxies to assess the potential impact of misclassification from proxy responses.

### Descriptive characteristics

**Fig. 2:**
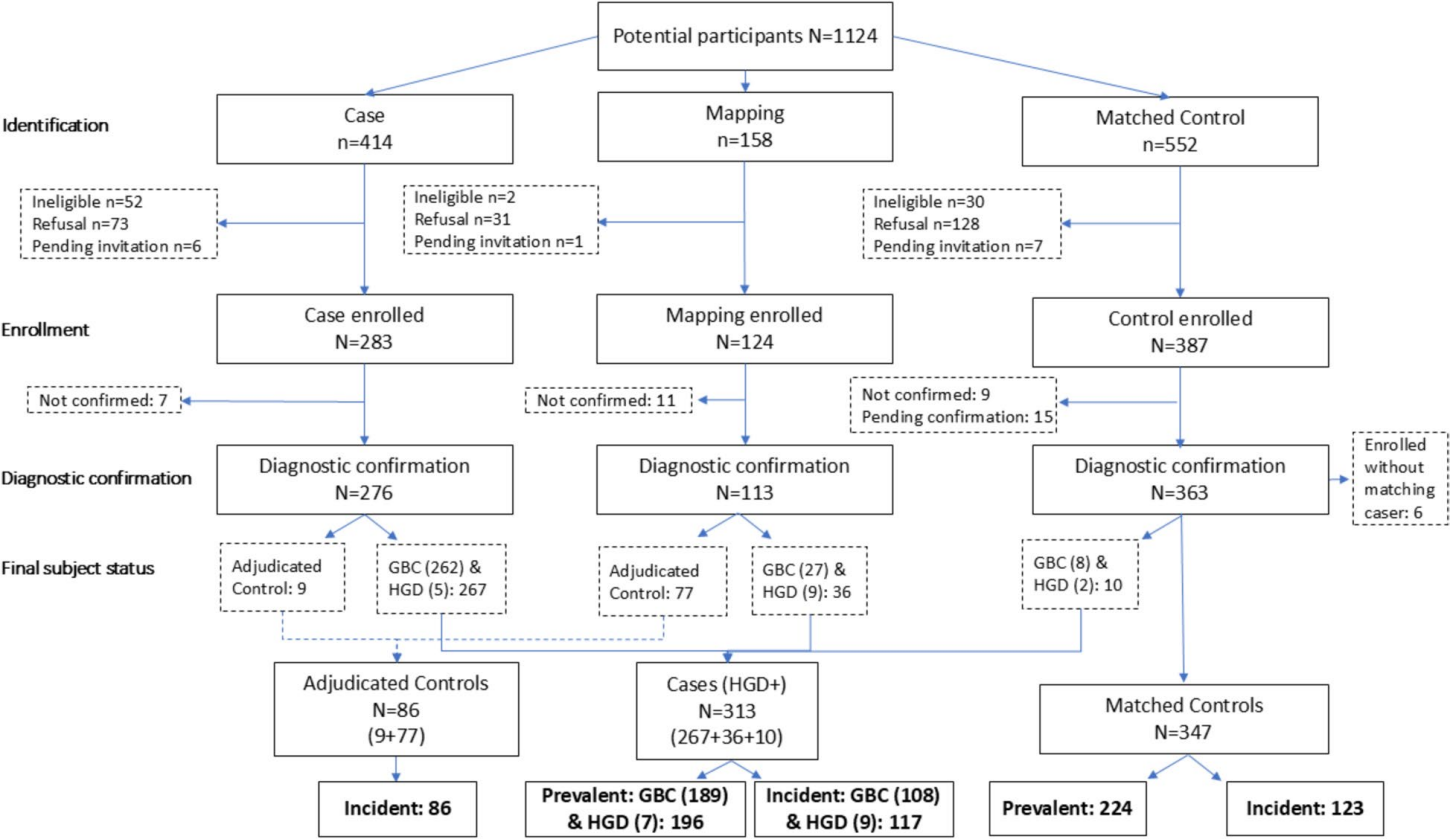
Enrollment flow chart for participants recruited from July 2022 through August 2025

Here we present data on participants who were recruited through August 2025. As of August 31, 2025, we had identified 1124 potential participants (Fig. 2). These potential participants included 414 potential cases identified through sources other than full mapping, 158 potential cases or adjudicated controls identified through full mapping, and 552 potential matched controls, of whom 283 (68.4%), 124 (78.5%), and 387 (70.1%) were enrolled, respectively. Case or non-case status was confirmed in 276 (66.7%), 113 (71.5%), and 363 (68.5%).

Of all eligible participants, 442 were eligible HGD + cases [(414 potential cases – 52 ineligible – 9 determined to be non-cases) + (158 potential mapping cases – 2 ineligible – 77 determined to be non-cases) + 10 participants recruited as matched controls later determined to be cases]. The participation rate among eligible HGD + cases was 70.8% (313/442). Of the 313 cases recruited, 196 were prevalent (189 GBC, 7 HGD) and 117 were incident cases (108 GBC, 9 HGD). Of the 297 enrolled GBC cases, 253 (85.2%) were identified incidentally through cholecystectomy, and 44 (14.8%) had a prior suspicion of cancer (clinical cases). GBC diagnosis was based on histopathological confirmation (i.e., through review of cholecystectomized tissue or biopsy) for 280 (94.3%) cases and on clinical history and imaging alone for 11 (3.7%).

We have also been successful recruiting controls. Of all eligible participants, 512 were eligible controls (552 potential controls – 30 ineligible – 10 later determined to be cases). The participation rate (controls who accepted participation/eligible controls) was 67.8% (347/512). Currently, 224 controls have been matched to 196 prevalent HGD + cases (100% of prevalent cases have at least one matched control), and 123 controls have been matched to 117 incident cases (98.3% of incident cases have at least one matched control).

We collected epidemiologic data through questionnaires from 306 (98%) of HGD + cases, 224 (100%) of prevalent controls, and 123 (100%) of incident controls (Table 1). We used proxies for 96 (49%) of the prevalent cases and 21 (9.4%) of the prevalent controls (no incident cases or controls had proxies). To date, we have collected blood from 98 (50%) prevalent cases, 111 (95%) incident cases, 200 (89.3%) prevalent controls, and 118 (96%) incident controls. Eight (1%) additional individuals [4 (1%) cases, 4 (1%) controls] did not provide blood but provided Oragene saliva samples as a source of germline DNA. The median time from blood collection to processing was 111 min. In addition, we have collected formalin fixed, paraffin embedded (FFPE) blocks from 4 (1%) cases and 86 (25%) controls. We also have 1634 digital H&E slide images from 20 (7%) cases and 54 (16%) controls.

**Table 1:** Collection of questionnaire data and biospecimens in cases and controls from the Chile BiLS case–control study

	Prevalent				Incident			
	HGD + cases (*N* = 196)		Matched Controls (*N* = 224)		HGD + cases (*N* = 117)		Matched Controls (*N* = 123)	
	N	%		%	N	%	N	%
Questionnaire	195	99.5%	224	100.0%	111	94.9%	123	100.0%
Proxy	96	49.0%	21	9.4%	0	0.0%	0	0.0%
Source of germline DNA	98	50.0%	201	89.7%	114	97.4%	121	98.4%
Blood	98	50.0%	200	89.3%	111	94.9%	118	95.9%
Oragene saliva sample	1	0.5%	1	0.4%	3	2.6%	3	2.4%
At least one pathology sample	10	5.1%	0	0.0%	24	20.5%	86	69.9%
FFPE blocks	1	0.5%	0	0.0%	3	2.6%	86	69.9%
Biopsies	9	4.6%	0	0.0%	21	17.9%	0	0.0%
At least one digital H&E slide image	2	1.0%	0	0.0%	18	15.4%	54	43.9%
Gallstones	0	0.0%	0	0.0%	1	0.9%	5	4.1%

The age distribution looks similar across enrolled prevalent and incident cases and controls except for incident HGD + cases, which included a larger proportion of people in the 50–54 age group than the older age groups (Table 2). Cases tend to be more likely to report Mapuche ancestry (40% prevalent, 41% incident) than controls (30% prevalent, 25% incident) and to have fewer years of education (71% and 61% prevalent and incident cases with 0–8 years of education vs. 67% and 48% of prevalent and incident controls). Interestingly, a notable proportion of both cases and controls (21%−20%) reported never having biliary colic, suggesting they had asymptomatic gallstone disease at the time of diagnosis.

**Table 2:** Sociodemographic, epidemiologic, and clinical characteristics of Chile BiLS case–control participants recruited from July 2022 through August 2025

	Prevalent*						Incident^†^					
	HGD + cases (*N* = 196)		HGD + matched controls (*N* = 224)		Adjudicated controls (*N* = 3)		HGD + cases (*N* = 117)		HGD + matched controls (*N* = 123)		Adjudicated (*N* = 104)	
	N	%	N	%	N	%	N	%	N	%	N	%
Age (years)												
< 50	17	8.7%	18	8.0%	0	0.0%	15	12.8%	17	13.8%	38	36.5%
50–54	13	6.6%	14	6.3%	1	33.3%	19	16.2%	20	16.3%	14	13.5%
55–59	18	9.2%	20	8.9%	0	0.0%	11	9.4%	12	9.8%	12	11.5%
60–64	28	14.3%	33	14.7%	0	0.0%	15	12.8%	13	10.6%	16	15.4%
65–69	42	21.4%	41	18.3%	0	0.0%	15	12.8%	13	10.6%	7	6.7%
70–74	30	15.3%	39	17.4%	0	0.0%	20	17.1%	23	18.7%	6	5.8%
75 +	48	24.5%	59	26.3%	2	66.7%	22	18.8%	25	20.3%	11	10.6%
Missing or unknown	0	0.0%	0	0.0%	0	0.0%	0	0.0%	0	0.0%	0	0.0%
Sex												
Female	155	79.1%	176	78.6%	2	66.7%	87	74.4%	91	74.0%	75	72.1%
Male	41	20.9%	48	21.4%	1	33.3%	30	25.6%	32	26.0%	29	27.9%
Ethnicity§												
Chilean/Latino	116	59.2%	155	69.2%	3	100.0%	61	52.1%	91	74.0%	66	63.5%
Mapuche	79	40.3%	68	30.4%	0	0.0%	48	41.0%	31	25.2%	36	34.6%
European	0	0.0%	1	0.4%	0	0.0%	0	0.0%	0	0.0%	1	1.0%
Other	0	0.0%	0	0.0%	0	0.0%	2	1.7%	1	0.8%	1	1.0%
Missing or unknown¶	1	0.5%	0	0.0%	0	0.0%	6	5.1%	0	0.0%	0	0.0%
Education (years completed)												
0–8 years	139	70.9%	151	67.4%	1	33.3%	71	60.7%	59	48.0%	42	40.4%
9–12 years	43	21.9%	60	26.8%	2	66.7%	34	29.1%	44	35.8%	40	38.5%
≥ 13 years	10	5.1%	13	5.8%	0	0.0%	6	5.1%	20	16.3%	21	20.2%
Missing or unknown¶	4	2.0%	0	0.0%	0	0.0%	6	5.1%	0	0.0%	1	1.0%
Health coverage												
None	1	0.5%	0	0.0%	0	0.0%	0	0.0%	0	0.0%	1	1.0%
FONASA Group A	79	40.3%	83	37.1%	1	33.3%	38	32.5%	40	32.5%	38	36.5%
FONASA Group B	63	32.1%	101	45.1%	1	33.3%	46	39.3%	54	43.9%	29	27.9%
FONASA Group C	15	7.7%	10	4.5%	0	0.0%	9	7.7%	9	7.3%	11	10.6%
FONASA Group D	10	5.1%	11	4.9%	0	0.0%	9	7.7%	9	7.3%	13	12.5%
FONASA (group unknown)	19	9.7%	17	7.6%	1	33.3%	6	5.1%	8	6.5%	6	5.8%
Other health coverage	5	2.6%	2	0.9%	0	0.0%	2	1.7%	3	2.4%	5	4.8%
Missing or unknown¶	4	2.0%	0	0.0%	0	0.0%	7	6.0%	0	0.0%	1	1.0%
Cigarette smoking status||												
Never	114	58.2%	142	63.4%	1	33.3%	60	51.3%	61	49.6%	54	51.9%
Former	57	29.1%	61	27.2%	1	33.3%	40	34.2%	35	28.5%	35	33.7%
Current	24	12.2%	21	9.4%	1	33.3%	11	9.4%	27	22.0%	15	14.4%
Missing or unknown¶	1	0.5%	0	0.0%	0	0.0%	6	5.1%	0	0.0%	0	0.0%
Alcohol drinking status**												
Never	32	16.3%	34	15.2%	1	33.3%	13	11.1%	11	8.9%	17	16.3%
Former	14	7.1%	26	11.6%	0	0.0%	12	10.3%	15	12.2%	10	9.6%
Current	148	75.5%	163	72.8%	2	66.7%	86	73.5%	97	78.9%	77	74.0%
Missing or unknown¶	2	1.0%	1	0.4%	0	0.0%	6	5.1%	0	0.0%	0	0.0%
Pesticide exposure (ever exposed)												
No	83	42.3%	174	77.7%	2	66.7%	82	70.1%	100	81.3%	84	80.8%
Yes	16	8.2%	29	12.9%	1	33.3%	29	24.8%	23	18.7%	20	19.2%
Proxy, not asked	96	20.0%	21	9.4%	0	0.0%	0	0.0%	0	0.0%	0	0.0%
Missing or unknown¶	1	0.5%	0	0.0%	0	0.0%	6	5.1%	0	0.0%	0	0.0%
Diabetes (ever diagnosed)												
No	162	82.7%	145	64.7%	3	100.0%	85	72.6%	85	69.1%	83	79.8%
Yes	32	16.3%	78	34.8%	0	0.0%	26	22.2%	37	30.1%	21	20.2%
Missing or unknown¶	2	1.0%	1	0.4%	0	0.0%	6	5.1%	1	0.8%	0	0.0%
Cardiovascular disease (ever diagnosed)††												
No	177	90.3%	196	87.5%	3	100.0%	107	91.5%	117	95.1%	95	91.3%
Yes	18	9.2%	26	11.6%	0	0.0%	4	3.4%	5	4.1%	9	8.7%
Missing or unknown	1	0.5%	2	0.9%	0	0.0%	6	5.1%	1	0.8%	0	0.0%
Hepatitis (ever diagnosed)												
No	190	96.9%	212	94.6%	3	100.0%	110	94.0%	115	93.5%	96	92.3%
Yes	4	2.0%	11	4.9%	0	0.0%	1	0.9%	8	6.5%	8	7.7%
Missing or unknown¶	2	1.0%	1	0.4%	0	0.0%	6	5.1%	0	0.0%	0	0.0%
Personal history of cancer, any												
No	179	91.3%	204	91.1%	3	100.0%	105	89.7%	113	91.9%	97	93.3%
Yes	15	7.7%	19	8.5%	0	0.0%	6	5.1%	10	8.1%	7	6.7%
Missing or unknown¶	2	1.0%	1	0.4%	0	0.0%	6	5.1%	0	0.0%	0	0.0%
Previous gallstone diagnosis												
No	41	20.9%	24	10.7%	0	0.0%	22	18.8%	6	4.9%	7	6.7%
Yes	150	76.5%	200	89.3%	3	100.0%	88	75.2%	117	95.1%	97	93.3%
Missing or unknown¶	5	2.6%	0	0.0%	0	0.0%	7	6.0%	0	0.0%	0	0.0%
Biliary colic (ever experienced intense epigastric pain lasting > 30 min)												
No	50	25.5%	43	19.2%	0	0.0%	22	18.8%	28	22.8%	22	21.2%
Yes	143	73.0%	181	80.8%	3	100.0%	89	76.1%	95	77.2%	82	78.8%
Missing or unknown§	3	1.5%	0	0.0%	0	0.0%	6	5.1%	0	0.0%	0	0.0%
Body mass index (BMI, kg/m2)												
Underweight (< 18.5)	0	0.0%	0	0.0%	0	0.0%	2	1.7%	0	0.0%	1	1.0%
Normal weight (18.5–24.9)	14	7.1%	16	7.1%	0	0.0%	26	22.2%	13	10.6%	13	12.5%
Overweight (25–29.9)	39	19.9%	70	31.3%	3	100.0%	38	32.5%	43	35.0%	35	33.7%
Obese I (30–34.9)	29	14.8%	73	32.6%	0	0.0%	30	25.6%	48	39.0%	37	35.6%
Obese II (35–39.9)	13	6.6%	29	12.9%	0	0.0%	12	10.3%	13	10.6%	13	12.5%
Obese III (> 40)	1	0.5%	15	6.7%	0	0.0%	2	1.7%	4	3.3%	4	3.8%
Proxy, not asked	96	49.0%	21	9.4%	0	0.0%	0	0.0%	0	0.0%	0	0.0%
Missing or unknown¶	4	2.0%	0	0.0%	0	0.0%	7	6.0%	2	1.6%	1	1.0%

^†^Incident participants were diagnosed or had cholecystectomy on or after July 19, 2022

^‡^Self-reported via risk factor questionnaire

^§^Mapuche: Ethnic group indigenous to Chile’s southern region; Other = Aymara/Quechua, Easter-Islander, or another ethnicity

^¶^Missing or unknown: Includes missing data and response “Participant doesn’t know / doesn’t respond.”

^#^ Health coverage: FONASA = Fondo Nacional de Salud (Chile’s public health insurance system). FONASA Groups A-D are hierarchical classifications used to determine the proportion of healthcare costs covered by the government, based on taxable individual income. FONASA Group A has the lowest income level and receives the highest level of governmental coverage of healthcare costs. Other health coverage = Governmental health coverage provided specifically for the Chilean armed forces or police service, private insurance, or other insurance

||Cigarette smoking status: Never = < 100 cigarettes smoked over lifetime; Former = Not currently smoking, smoked ≥ 100 cigarettes over lifetime; Current = Currently smoking, smoked ≥ 100 cigarettes over lifetime

^*^Prevalent participants were diagnosed or had cholecystectomy from January 1, 2016, through July 18, 2022

^**^Alcohol drinking status: Never = Never has consumed alcohol; Former = Does not currently consume alcohol but has consumed it in the past; Current = Currently consumes alcohol

## Discussion

Chile has among the highest reported rates of GBC in the world [5] and high rates of gallstones as well [27], especially among individuals of Mapuche ancestry [28, 29]. The high rates of both GBC and its most important risk factor make Chile a good place to study the drivers of GBC. We have leveraged these factors by designing a case–control study to complement our on-going cohort study in the southern-central region of Chile, which has the highest risk for GBC. By enriching the number of GBC cases, this case–control study will increase our ability to understand the associations between risk factors and GBC among people who have gallstones. It will also improve our potential to identify markers for early detection or risk stratification strategies, which could be important for high-risk populations.

There are some limitations to consider. For example, prevalent cases diagnosed early in the 2016–2022 period and enrolled years later represent longer-term survivors, which might distort potential associations with aggressive disease. Evaluation of associations restricted to incident versus prevalent cases and controls can help inform our understanding of this potential bias in future analyses. In addition, approximately 50% of prevalent cases and 9% of prevalent controls used proxies. Differential misclassification might be possible, which can be assessed through sensitivity analyses excluding case–control pairs with proxies. Finally, adjudicated controls with suspicious lesion but benign pathology represent a different population than matched gallstone controls and should not be combined without careful consideration.

Although this study is not an intervention trial, it includes family-oriented elements of health education and screening within the study procedures. During the informed consent process, participants received information on biliary disease, associated risk factors, and general preventive measures, including healthy lifestyle behaviors. In addition, during home visits conducted by trained healthcare personnel, these topics were discussed in the context of the interview, often leading to informal engagement with family members present in the household. The study also offered abdominal ultrasound screening to up to two family members, providing an opportunity for early detection and clinical orientation. Furthermore, the study team remained available to address concerns and facilitate access to ultrasound evaluation for participants or their relatives when needed. These elements highlight the potential role of integrating family-oriented approaches in future prevention strategies.

This study is particularly valuable as the lessons we learn in Chile can also be applied elsewhere. For example, the National Health Service cholecystectomy waiting list in the United Kingdom is 12–27 weeks [30, 31]. There is a need to identify the patients most urgently in need of surgery [30]. Thus, identifying meaningful ways to prioritize the cholecystectomy waiting list has utility in other parts of the world. Furthermore, GBC is one of the cancers that is most often associated with obesity [32, 33]. Given the increasing impact of the global obesity epidemic [34], understanding the relationship between obesity and GBC could have important implications for other cancers and obesity-related conditions.

### Opportunities to collaborate

As a rare cancer, international collaborations are needed to move GBC research forward. Chile BiLS itself is a large, multidisciplinary, international collaboration, and we encourage and promote external collaborations. Our rigorous collection of detailed epidemiologic and clinical data, along with our growing biorepository, will provide a valuable framework for collaborative efforts. We especially value and encourage international collaborations to evaluate similarities and differences across high- and low-risk populations. Outside investigators should submit a request to the Chile BiLS Steering Committee for review and approval. To request guidelines for application, please email the Chile BiLS coordinator at ChileBILS@westat.com.

### Conclusion

To conclude, the Chile BiLS case–control study is an expansion of the Chile BiLS cohort that enriches the number of cases and enhances opportunities to identify important associations between risk factors and GBC among people with gallstones. It will also accelerate attempts to identify markers for early detection or risk stratification. Given the limited treatment options and high mortality due to GBC, this study will provide an important step forward.

## Supplementary Information

Supplementary Material 1: Supplemental Table 1. Abnormal ultrasound findings among participants’ family members leading to referral to health system hospitals for additional care from January 2023 through August 2025. Supplementary Table 2. Comparison of surveys applied to the participant and proxy.

## Data Availability

The dataset analyzed for this publication is available by contacting Dr. Catterina Ferreccio ([cferrecr@uc.cl](/cdn-cgi/l/email-protection#)) and Claudia Marco ([cmarco@uc.cl](/cdn-cgi/l/email-protection#)) upon reasonable request.

## Abbreviations

Chile BiLS: Chile Biliary Longitudinal Study
FFPE: Formalin-fixed, paraffin-embedded
GBC: Gallbladder cancer
HGD: High-grade dysplasia
HHHA: Hospital Dr. Hernán Henríquez Aravena
