# Supplementary material for: The Chile BiLS case–control study of gallbladder cancer
Source: Arch Public Health. 2026 Jun 6;84:182. doi: 10.1186/s13690-026-01977-1 (PMC13463823; doi:10.1186/s13690-026-01977-1)
Supplement: Supplementary file 1 [file 13690_2026_1977_MOESM1_ESM.docx]

**Supplemental Table 1.** Abnormal ultrasound findings among participants’ family members leading to referral to health system hospitals for additional care from January 2023 through August 2025.

| **Medical findings** | **n** |
| --- | --- |
| Steatosis liver disease | 18 |
| Liver cyst | 7 |
| Liver tumor | 3 |
| Liver cancer | 4 |
| Pancreatic cyst | 2 |
| Gallbladder polips | 2 |
| Multiple cholelithiasis | 2 |
| Chronic pancreatitis | 2 |
| Gallbladder cancer | 1 |
| Hepatitis | 1 |
| Chronic liver damage | 1 |
| Kidney cyst | 1 |
| Kidney nodule | 1 |
| Pancreatic tumor | 1 |
| Kidney stones | 1 |
| Kidney tumor | 1 |
| **Total** | **48** |

| **Supplementary Table 2.** Comparison of surveys applied to the participant and proxy | | | |  |
| --- | --- | --- | --- | --- |
|  |  |  |  |  |
| **Assessment dimension** | **Variables** | **Participant** | **Proxy** |  |
| **Socio  demographics** | Sex | ✓ | ✓ |  |
|  | Marital status | ✓ | ✓ |  |
|  | Country of birth | ✓ | ✓ |  |
|  | Ethnicity | ✓ | ✓ |  |
|  | Educational level | ✓ | ✓ |  |
|  | Health insurance system | ✓ | ✓ |  |
| **Medical  history** | Weight history | ✓ | ✓ |  |
|  | Diabetes mellitus | ✓ | ✓ |  |
|  | High blood pressure | ✓ | ✓ |  |
|  | Hepatitis | ✓ | ✓ |  |
|  | Typhoid/paratyphoid fever | ✓ | ✓ |  |
|  | Ulcerative colitis | ✓ | ✓ |  |
|  | Pancreatitis | ✓ | ✓ |  |
|  | Crohn's disease | ✓ | ✓ |  |
|  | Appendicitis | ✓ | ✓ |  |
|  | Thyroid disease | ✓ | ✓ |  |
|  | Liver cirrhosis | ✓ | ✓ |  |
|  | Other liver disease | ✓ | ✓ |  |
|  | Other cancers besides gallbladder cancer | ✓ | ✓ |  |
| **Gastrointestinal  health** | Prolonged upper abdominal pain without diarrhea | ✓ | ✓ |  |
|  | Jaundice | ✓ | ✓ |  |
|  | Acute cholecystitis | ✓ | ✓ |  |
|  | Gallstone disease | ✓ | ✓ |  |
| **Cardiovascular  health** | Heart attack or coronary artery disease | ✓ | ✓ |  |
|  | Stroke | ✓ | ✓ |  |
|  | Blood pressure intake | ✓ | ✓ |  |
| **Gynecologic and obstetric history** | Menstrual status | ✓ |  |  |
|  | Hormonal treatment: hormonal contraceptives/Hormone replacement | ✓ | ✓ |  |
|  | Pregnancy history | ✓ | ✓ |  |
|  | Months of breastfeeding with each of your children | ✓ |  |  |
|  | Lithiasis during pregnancies | ✓ | ✓ |  |
|  | Cholestasis of pregnancy | ✓ |  |  |
| **COVID-19  History** | Dg, severity, hospitalization, vaccinations | ✓ | ✓ |  |
| **Medications** | Regular use of antibiotics, corticosteroids, proton pump inhibitors, aspirin or other NSAIDs, statins or any type of alternative medicine or supplements. | ✓ | ✓ |  |
| **Family history  of biliary disease** | Blood-related family history of gallstones, liver cancer, gallbladder cancer, other cancers, or cholecystectomy | ✓ | ✓ |  |
| **Food consumption frequency** | Fresh green or red chili, or red chili paste | ✓ | ✓ |  |
|  | Red meat (low or high fat) | ✓ |  |  |
|  | Fried foods | ✓ | ✓ |  |
|  | Corn kernels, peanuts, nuts, and other dried fruits | ✓ |  |  |
|  | Sugary drinks or juices | ✓ |  |  |
|  | Cups of tea, coffee, mate, or herbal water per day | ✓ | ✓ |  |
| **Smoking** | Lifetime Smoking History (100 or more cigarettes) | ✓ | ✓ |  |
|  | Current Smoking Status (at diagnosis) | ✓ | ✓ |  |
|  | Average Cigarettes Smoked per Day in the Last Month | ✓ |  |  |
|  | Average Cigarettes Smoked per Day During Regular Smoking Years | ✓ |  |  |
|  | Household Smoking History | ✓ | ✓ |  |
|  | Exposure to Secondhand Smoke at Work or Study | ✓ | ✓ |  |
| **Alcohol  consumption** | Alcohol Consumption History: First Alcohol Consumption, Regular Drinking Start Age, and Age When Stopped Drinking | ✓ | ✓ |  |
|  | Average Glasses Consumed per Occasion in the Last 5 Years (Wine, Beer, and Other Spirits) | ✓ |  |  |
|  | Alcohol Consumption at the Time of Gallbladder Cancer Diagnosis | ✓ |  |  |
| **Employment  history** | Paid Job History (more than 6 months) | ✓ | ✓ |  |
|  | Paid Job in the Last Year | ✓ | ✓ |  |
|  | Longest Job and Job Description | ✓ | ✓ |  |
| **Pesticide  exposure** | Lifetime Work with Pesticides (Agriculture, Weeds, Plants) | ✓ |  |  |
|  | Total Years Worked in Agriculture Pesticide Use | ✓ |  |  |
|  | Types of Crops Worked With | ✓ |  |  |
|  | Lifetime Work with Pesticides (Insects or Animals) | ✓ |  |  |
|  | Total Years Worked in Insect/Animal Pesticide Use | ✓ |  |  |
|  | Lifetime Work with Pesticides (Housing or Gardens) | ✓ |  |  |
|  | Total Years Worked in Housing/Garden Pesticide Use | ✓ |  |  |
|  | Personal Handling or Application of Pesticides | ✓ |  |  |
| **Physical  activity** | Average Time Spent Sitting or Lying Down per Day (Work, Travel, Leisure) | ✓ |  |  |
|  | Average Time Spent Sitting (5 years ago) | ✓ |  |  |
|  | Physical Activity or Sports Participation History | ✓ | ✓ |  |
|  | Types of Regular Physical Activities or Sports | ✓ | ✓ |  |
|  | Total Years of Regular Physical Activity or Sports Participation | ✓ |  |  |
